# Extraction of the Polyurethane Layer in Textile Composites for Textronics Applications Using Optical Coherence Tomography

**DOI:** 10.3390/polym10050469

**Published:** 2018-04-25

**Authors:** Jarosław Gocławski, Ewa Korzeniewska, Joanna Sekulska-Nalewajko, Dominik Sankowski, Ryszard Pawlak

**Affiliations:** 1 Institute of Applied Computer Science, Lodz University of Technology, 18/22 Stefanowskiego Street, Lodz 90-924, Poland; jsekulska@kis.p.lodz.pl (J.S.-N.); dsan@kis.p.lodz.pl (D.S.); 2 Institute of Electrical Engineering Systems, Lodz University of Technology, 18/22 Stefanowskiego Street, Lodz 90-924, Poland; ewakorz@matel.p.lodz.pl (E.K.); ryszard.pawlak@p.lodz.pl (R.P.)

**Keywords:** composite fabric, polyurethane layer, optical coherence tomography, thickness map, 2D polynomial approximation, 2D spline smoothing

## Abstract

This article presents a new method for the extraction and measurement of the polyurethane layer of Cordura textile composites using optical coherence tomography. The knowledge of coating layer properties in these composites is very important, as it affects mechanical parameters such as stiffness and bending rigidity. Unlike microscopic measurements, which require cross-section samples of the material, the proposed approach is non-invasive. The method is based on detecting the top and bottom boundaries of the polyurethane layer in Optical Coherence Tomography (OCT) images using image processing methods, namely edge enhancement filtering, thresholding and spline smoothing. The cover layer measurement results obtained from a three-dimensional OCT image of the composite fabric are presented as the thickness maps. The average values of the layer thicknesses measured with the OCT method for four types of Cordura showed a high correlation with the results obtained from microscopic measurements (Pearson correlation coefficient r=0.9844), which confirms the accuracy of the OCT method.

## 1. Introduction

**Figure 1:**
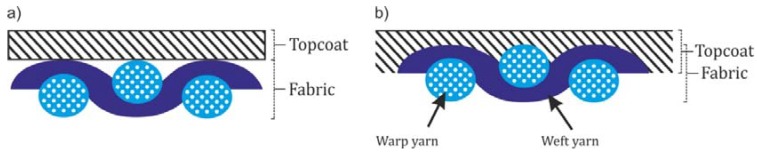
Two ways of applying a polymer layer to fabrics: (**a**) lamination; (**b**) coating.

Textile composites are made of polymer materials with textile reinforcements. The textile substrate of the composite, which is typically a woven, knitted or non-woven textile fabric, is combined with a thin, flexible film made of a natural or synthetic polymeric substance (thermoplastic or thermoset) [1,2,3,4]. This film can be applied by either coating or laminating. Both techniques are efficient and functional, although each is used for specific purposes, depending on the textile materials and the required finish. The main difference between coating and laminating lies in the method by which the topcoat is applied to the fabric used as the base layer (Figure 1). In the case of lamination, both the substrate and the top layer are in a solid state. Bonding usually takes place as a result of pressure and heating, which causes a slight softening of the laminating layer. The bond between the layers is much weaker than with coating, and sometimes delamination occurs. In the case of coating, the polymer component is in a liquid phase when it is applied to the textile layer. The coating penetrates the surface layer into the substrate structure, providing better bonding between the components. Applying a polymer layer to the top surface of a material makes it stiffer, especially in the case of coating, which fills the space between the threads of the weft and warp. The bending rigidity and tensile modulus also increase [5,6]. The influence of warp density on the physical–mechanical properties of the material is significant, but coating can provide an up to twofold increase in mechanical strength [6,7]. The mechanical strength of the material depends on the thickness of the polymer covering layer, as well as on its chemical composition and the content of polyethylene glycol (PEG) [8]. The mechanical properties of textile composites also depend on the surface properties of the polymer film, on the degree to which it penetrates the textile layer and on the structural characteristics of the textile reinforcement [7,9].

Textile composites have a wide range of military, medical, industrial and consumer applications, including in military vehicles and weaponry, as medical substrates, as industrial fabrics and in outdoor clothing [1,8,10,11,12,13], where high mechanical resistance (e.g. tensile strength and resistance to abrasion) is a primary consideration [9]. Composite materials with polymer coatings are also very important for covering surfaces with an anti-bacterial, anti-fungal, anti-allergic or anti-static layers [5,10,14,15,16,17]. Recently, laminated textiles have been shown to provide an excellent substrate for creating flexible passive electronic elements and circuits using Physical Vapor Deposition (PVD) technology and laser patterning [18,19]. Due to the smoothness of fabrics with polymer coatings, electro-conductive metal layers deposited on textile composites preserve good conductivity over large surface areas. The use of a clear layer of polyurethane also can also guarantee high conductivity between conductive metallic layers. Moreover, a polymer surface layer can help protect against bending and make conducting layers more resistant to stretching stresses [18]. Various types of sensor can be embedded in highly durable layers of polymer, which provide shielding against electromagnetic and radio frequency interference (EMI/RFI) [10,20,21,22]. Before a laminated or coated composite material is used for a particular application, it is necessary to determine the thickness of the surface layer. Commercially available textile composites have a very diverse range of polymer thicknesses. However, the nominal thickness values in technical data sheets almost always refer to the total thickness of the fabric. The thickness of the polymer is not included. One example is the composite material known under the trade name Cordura, which has a surface layer of polyurethane [12,23]. The patent is owned by DuPont, and Invista has a license to manufacture and sell the product. The process of synthesis and the chemical properties of polyurethane are described in [24,25,26,27].

The mechanical strength of Cordura is many times greater than the mechanical strength of cotton or polyester, and several times greater than that of nylon. Quick drying and low weight, this resilient material is used in the fabrication of outdoor equipment, including equipment used by the army and emergency services. Accurate values for the total thicknesses of fabrics under specified pressures are easily obtained by standard methods, such as ISO 5084:1996 and ASTM D374–99 (2004). The thicknesses of entire composites or composite layers can also be measured using optical and scanning microscopy (SEM). Spectral methods, such as Fourier transform infrared spectroscopy (FTIR), X-ray photo-electron spectroscopy (XPS), atomic force microscopy (AFM) and time-of-flight secondary ion mass spectrometry (ToF-SIMS), can be also applied for the analysis of functionalized polymer surfaces [14,28,29,30]. However, all these methods require cross-section samples of the material. In this paper, we propose a new, non-invasive method for the evaluation of polymer layer thickness, using optical coherence tomography (OCT). With OCT, it is possible to make visual inspections of materials with non-uniform structures that are penetrable by infrared radiation to a depth of 1 to 2 mm. Recently, different OCT systems have been used for the structural characterization of polymers and composite materials [31,32], including thickness measurements of non-woven composite cover layers [33]. The aim of our research was to develop a method for the automatic detection and estimation of the thicknesses of the surface layers of textile composites. The method was tested on Cordura with different degrees of surface area coverage and varying degrees of integration between the polyurethane layer and the base material. The method presented in this work is a new, non-invasive approach to the infrared measurement of semi-translucent polymer layers in composite textiles. Our analysis of the surface parameters of Cordura further allows an assessment of its suitability as a carrier of metallic layers and for other industrial applications.

## 2. Material and Methods

### 2.1. Cordura Samples

Analysis was performed on four types of Cordura material, made of textured nylon fibers with a thin polyurethane film visible in OCT imaging. These materials are referred to in the text according to the scheme described in Table 1.

To characterize the investigated composite, infrared spectra were measured with a BIO-RAD FT-IR 175C (Bio-Rad Corporation, Hercules, CA, USA) spectrophotometer in the wavenumber range from 400 to 4000 cm^−1^. In all textile composites, the FTIR spectra of the polymer layer exhibited bands of about 3330 cm^−1^ (stretching N–H bonds), 2845 cm^−1^ (stretching C–H bonds), 1730 cm^−1^, 1600 cm^−1^ and 1520 cm^−1^ (stretching C=O and N–H bonds), which allowed the identification of the material as polyurethane. The chosen FTIR analysis, performed in an upper polymer layer, is shown in Figure 3 for Cordura Yellow and Cordura Camouflage.

Additionally, the test samples were subjected to tensile tests. The obtained results are presented in Figure 4. The graphs show the high strength of all materials in terms of tensile stress. All materials were characterized by high flexibility; the deformation limit for the tested samples was 120 to 155%.

### 2.2. Measurement with Microscope Images

**Figure 2:**
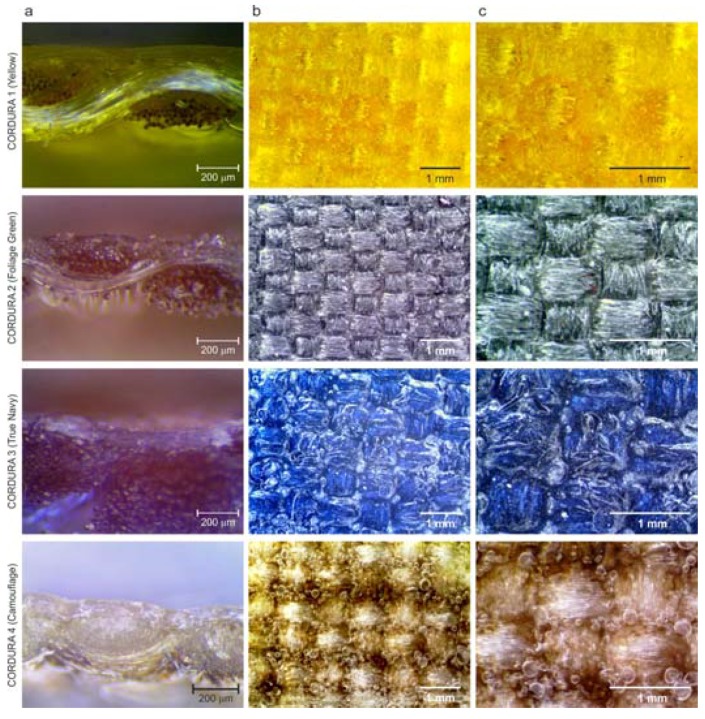
Cross-sections (**a**) and surfaces (**b**,**c**) of examined Cordura materials visible under a Delta Optical SZ–630T bright-field microscope; images obtained using ×100, ×20 and ×40 magnifying lenses respectively.

**Figure 3:**
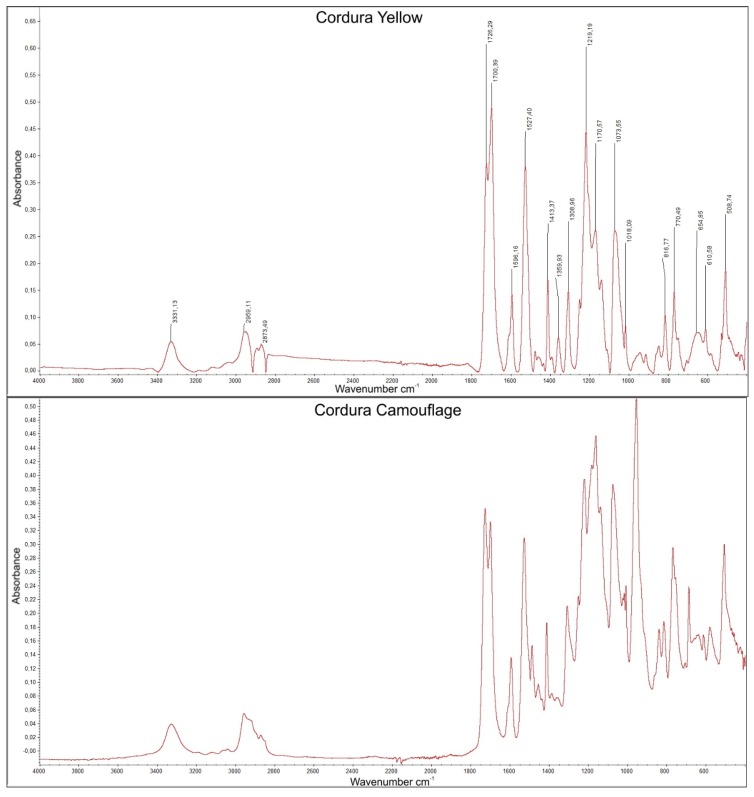
FTIR spectra for Cordura Yellow and Cordura Camouflage.

**Figure 4:**
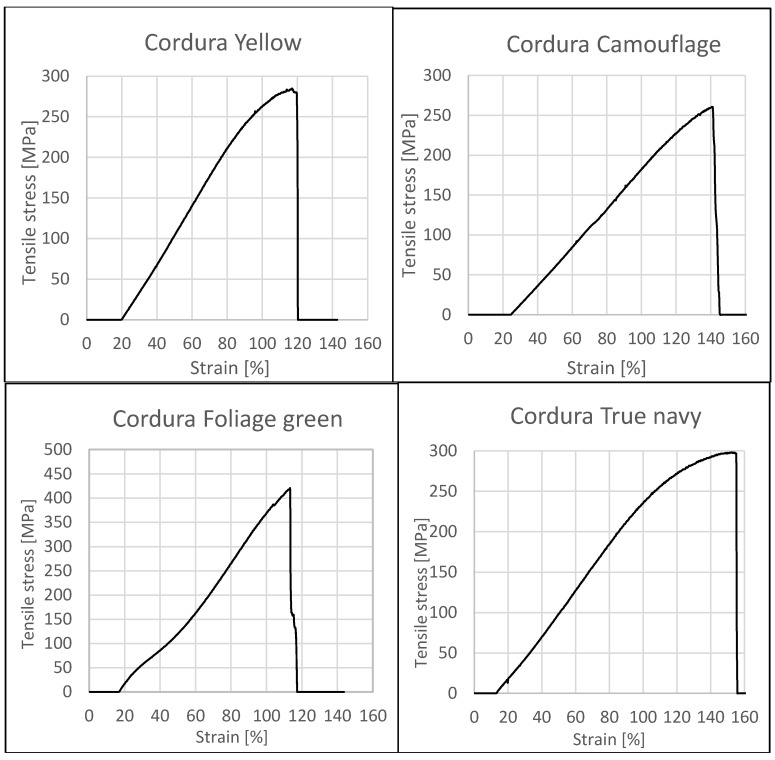
Stress–strain curve for the investigated composites.

**Figure 5:**
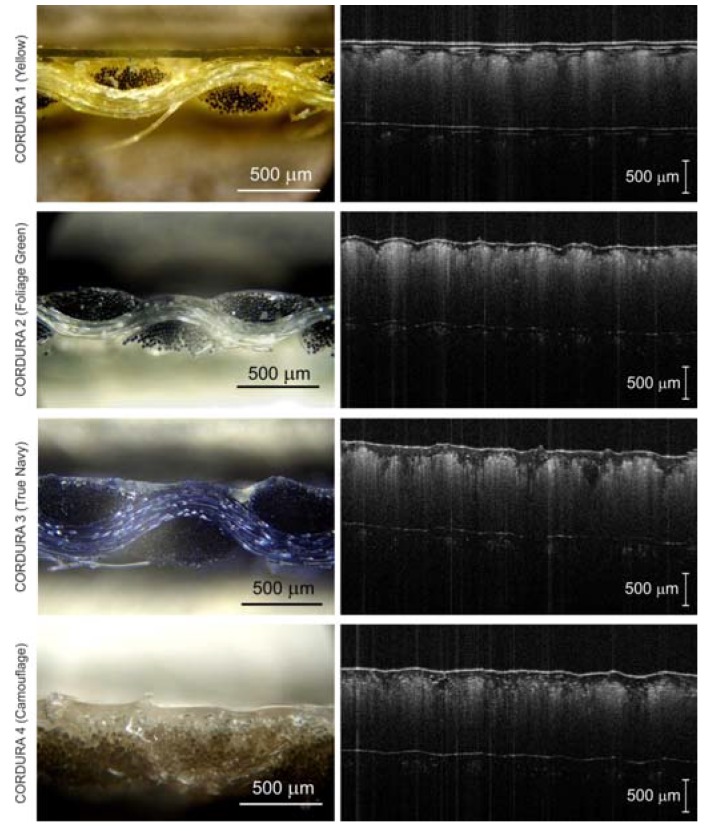
Cross-section images of Cordura materials made using Neophot 21 dark-field microscope with an objective magnification of ×80 or ×100 (left column); and corresponding OCT *B*-scans of the same Cordura samples obtained with Spark OCT-1300 system (right column).

**Figure 6:**
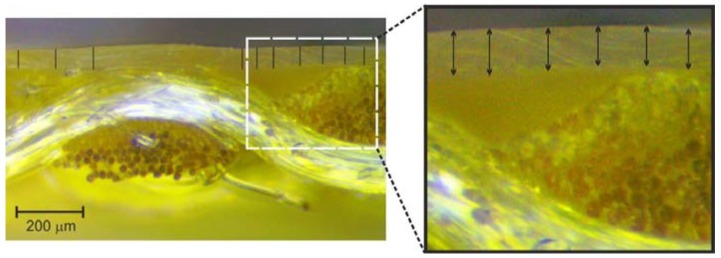
Illustration of the idea of measuring the thickness of a Cordura polyurethane layer in bright-field microscopic images using the line tool of Adobe Photoshop software.

Cross-section samples of the Cordura materials were studied using both dark-field and bright-field stereoscopic microscopes (Neophot 21, Carl-Zeiss, Jena, Germany) and Delta Optical SZ–630T (Delta Optical, Warsaw, Poland) respectively) using ×60, ×80 and ×100 magnifying lenses (Figure 2 and Figure 5). The surfaces of the Cordura materials were further imaged using a bright-field Delta Optical SZ–630T microscope with ×20 and ×40 magnifying lenses (Figure 2). The line tool in Adobe Photoshop was used as a ruler to measure the thickness of the polyurethane layers in the microscopic images (Figure 6). A series of 12 measurements (lines) were taken for each type of material in a random selection of places where the polyurethane layer was clearly visible. As can be seen in Figure 2 and Figure 5, the images of the outer polyurethane layers of the Cordura cross-sections presented significant differences. Only in the case of Cordura 1 (Figure 5) did the top polyurethane layer have an almost constant thickness as a result of the laminating process, with characteristic voids under the surface of the polyurethane layer. The other materials (Cordura 2, Cordura 3 and Cordura 4 in Figure 5) exhibited the effects of the coating process. The top polymer layers were of irregular thickness. The polyurethane layers had different degrees of smoothness. In Cordura 4, the polyurethane penetrated the warp and weft yarns. These differences could have had a significant impact on both the physical vapor deposition (PVD) of the metallic conductive layers on the Cordura surface and on the mechanical and electrical properties of any such conductive layers. However, obtaining a full, averaged image of the polyurethane layer, including its geometry and thickness, would require microscopic examination of many material cross-sections. Such microscopic examinations are tedious and do not provide sufficient accuracy. These problems can be solved by applying the new optical coherence tomography approach presented here.

### 2.3. Acquisition of OCT Images

**Figure 7:**
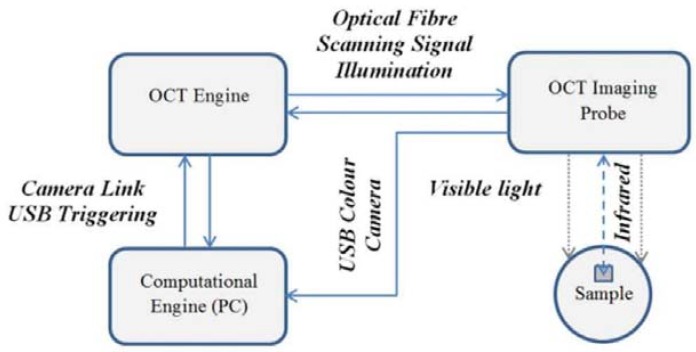
Block diagram of the Spark OCT 1300 nm system.

**Figure 8:**
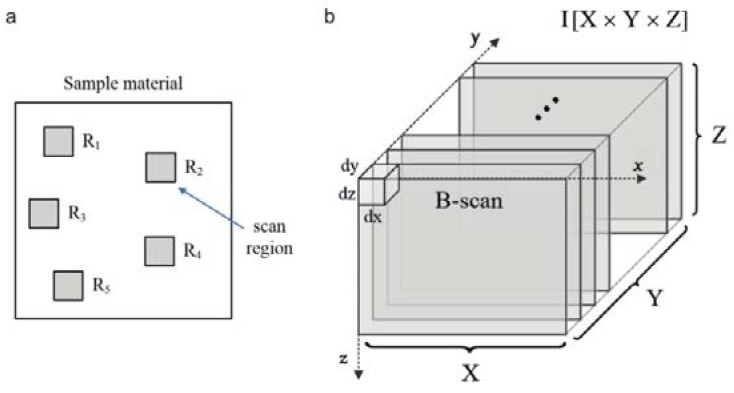
Acquiring images of the composite material samples: (**a**) random selection of imaging regions; (**b**) OCT imaging of region *R*_k_ as a stack of XZ planes (*B*-scans) acquired using a Spark OCT 1300 system; $R_{k},k\in \left[1,5\right]$—top view of a scanned image region; dx,dy,dz—dimensions of each voxel; X, Y, Z—image size of the region in voxels.

A Spark OCT-1300 system by Wasatch Photonics Inc. (Durham, NC, USA). It was used for the visualization of the subsurface structures in the composite fabrics [34]. A block diagram of the system is shown in Figure 7. The device consists of three modules. The OCT engine contains an interferometer, electronics, a spectrometer, the reference arm, and polarization and path length controllers. The Michelson’s interferometer is used to obtain the Fourier transforms from the interference signals using a spectrally scanning source. The depth can be calculated directly from the acquired Fourier-transform spectra, without movement of the reference arm. The OCT imaging probe contains a light source, scanning mirror, optics and a colour camera for creating fundus images of the scanned region. The computational engine (PC) includes a camera link card for the acquisition of data from the spectrometer and to receive synchronization triggers from the engine. Once the infrared laser light (around 1300 nm wavelength) has penetrated the material region, the reflected infrared signal is registered as a three-dimensional image array in the PC memory. The image is then saved by Wasatch Photonics firmware (Durham, NC, USA) as a multi-frame DICOM file for further processing. OCT images were acquired of randomly-selected disjunctive cuboid regions of the tested composite materials (Figure 8a). Each scanned region consisted of [X×Y×Z] voxels, of size [dx×dy×dz], where dx=4.58 μm, dy=4.64 μm, dz=5.4 μm (Figure 8b). The original scanning region of [512×512×1024] voxels was limited to *Z* = 800 voxels in each of the sample region images, which corresponded to a physical volume of around [4.70 mm×4.75 mm×4.32 mm] when the image of every second voxel is saved in directions *X* and *Y*. The limitation in *Z* direction allowed the removal of deep sample layers, which do not include any visible structures except for the mora of the air–material boundary. The material surface typically occurs at 20% of the *Z* range, which means that OCT scanning penetrates the composite fabric up to a depth of approximately 3.5 mm. The scanning process provides *B*-scan frames (XZ planes) in real time and composes a full 3D image from these frames in off-line mode, an operation that takes around 20 s.

### 2.4. Computing Environment

Each sample was scanned in 10 different regions $R_{k},k=1,\ldots ,10$, as shown in Figure 8a. This provided a total of 40 OCT images, which were trimmed in size to [512×512×800] voxels. The algorithm was executed using a computational engine working in the Spark OCT system (Figure 7). The PC was equipped with 64 GB RAM, two CPU units Intel(R) Xeon(R), E5–2695 v2 (Intel Corporation, Santa Clara, CA, USA) clocked at 2.40 GHz and 12 physical cores. Image filtering stages were implemented with the support of GPU parallel computing using NVidia CUDA technology [35]. The graphics card used for computing was the NVidia GeForce Quadro K6000 with 2880 CUDA cores (Nvidia Corporation, Santa Clara, CA, USA), computing capability 3.5 and 12GB DDR5 on-board RAM. The algorithm as a whole was developed and executed in a MATLAB 2015b environment, equipped with the Image Processing Toolbox [36]. The GPU–supported modules were built by NVidia and Visual Studio 2008 compilers [37] were used as MEX files.

## 3. Image Analysis

**Figure 9:**
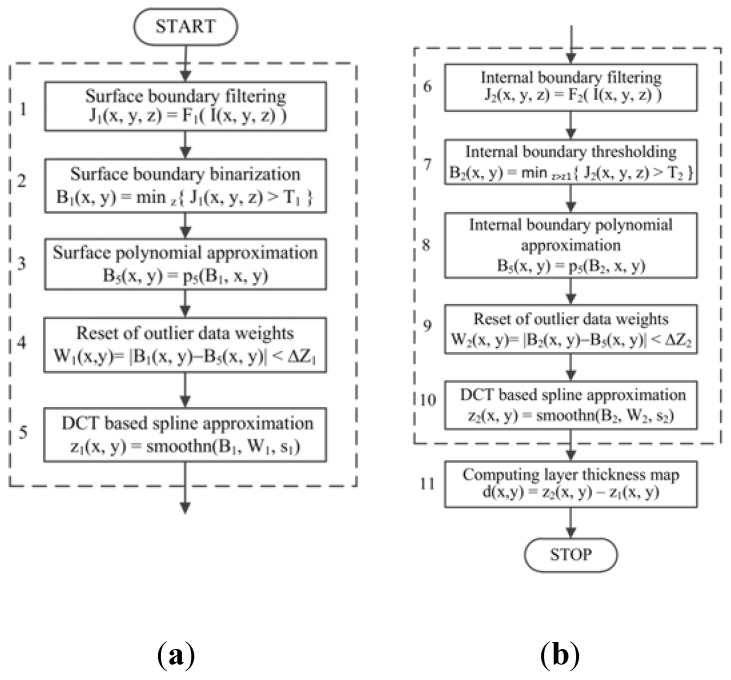
Algorithm flow for measurement of composite layer thickness in a single sample region: (**a**) surface approximation stage (1) ÷ (5); (**b**) internal boundary evaluation stage (6) ÷ (10); I(x,y,z)—the original image of the sample region $R_{k}\left[X\times Y\times Z\right]$ (Figure 8); J(x,y,z)—the filtered image with emphasized boundaries; $F_{1}\left(\cdot \right)$, $F_{2}\left(\cdot \right)$—boundary filtering procedures; *T*_1_, *T*_2_—the thresholds for boundary detection; *B*_1_—the set of surface boundary fragments (Figure 10a); *B*_2_—the set of internal boundary fragments (Figure 10c); $p_{5}\left(\cdot \right)$—fifth order polynomial approximation in the XY plane; smoothn(·)—DCT based spline approximation in the XY-plane; $s_{1},s_{2}>0$—the smoothing levels for spline approximations; *W*_1_, *W*_2_—Boolean data weight arrays; $z_{1}\left(x,y\right)$—the approximated 2D function of a surface boundary (Figure 10b); $z_{2}\left(x,y\right)$—the approximated 2D spline function of an inner boundary (Figure 10d); d(x,y)—the image of cover layer thickness (Figure 10e,f).

The proposed segmentation method extracts the cover layer boundaries from the OCT composite images and creates a layer thickness map. The OCT input image I[X×Y×Z] is loaded from a DICOM file prepared using the OCT firmware discussed in Section 2.3. The algorithm flow is illustrated in Figure 9 and consists of three main stages:

1. Detection and approximation of the composite surface boundary in the XY plane of the imaging region (Figure 9a).
2. Detection and approximation of the internal boundary of the composite cover layer in the XY plane of the imaging region (Figure 9b).
3. Evaluation of the thickness maps of the cover layer by subtraction of the approximated boundaries (Figure 9b).

The first steps in stages 1 and 2 (Figure 9 (1), Figure 9 (6)) include image preprocessing in the form of local filtering $F_{1}\left(\cdot \right)$ and $F_{2}\left(\cdot \right)$. OCT images are usually distorted by the strong speckle noise that always occurs during infrared short coherence laser scanning [38]. Typically, diffusion or median filters are used to enhance OCT images with a minimal loss of image detail [39,40]. $F_{1}\left(\cdot \right)$ and $F_{2}\left(\cdot \right)$ are the cascades of a local mean filter followed by a Laplacian of Gaussian (LoG) filter in the image space. The local mean filter (MF) is expressed by the formula:

(1) $$J\left(x,y,z\right)=\frac{1}{PQR}\sum_{u=-P/2}^{P/2}\sum_{v=-Q/2}^{Q/2}\sum_{w=-Q/2}^{Q/2}I\left(x+u,y+v,z+w\right),$$

where I(x,y,z) and I(x,y,z) represent values for the voxels (x,y,z) in the source and destination images, respectively; and the parameters *P*, *Q* and *R* denote the dimensions of a cuboid region surrounding the central voxel (x,y,z). 

The applied LoG filter in three dimensions follows the rule:

(2) $$JJ\left(x,y,z\right)=\frac{1}{PQR}\sum_{u=-U/2}^{U/2}\sum_{v=-V/2}^{V/2}\sum_{w=-W/2}^{W/2}I\left(x+u,y+v,z+w\right)\cdot K\left(u,v,w\right),$$

**Figure 10:**
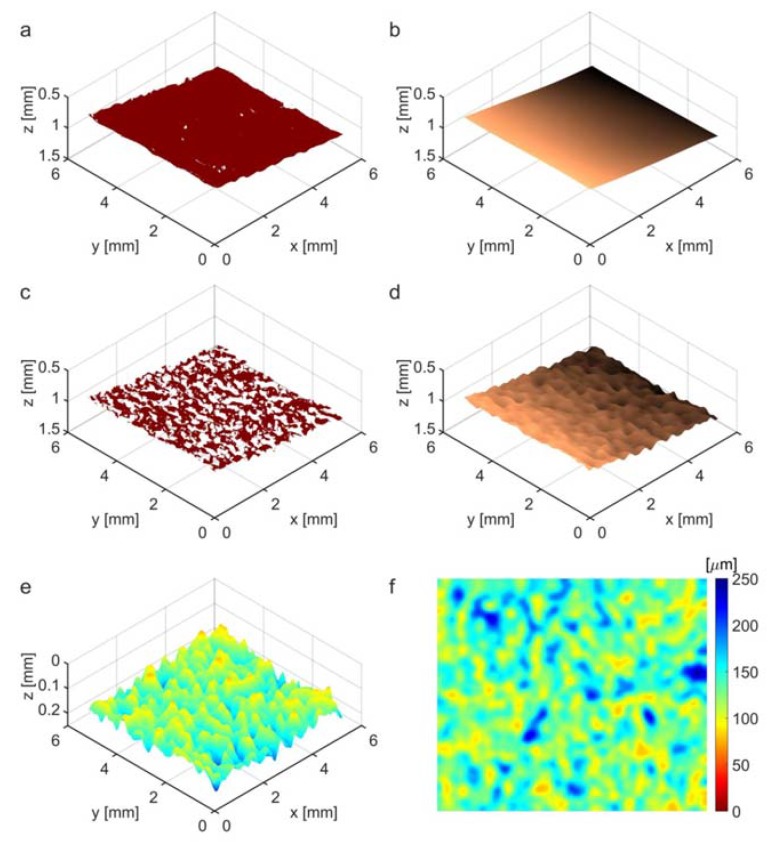
Illustration of selected stages in the Cordura image segmentation work-flow presented in Figure 9. (**a**) Cordura surface boundary; (**b**) Cordura surface approximated with fifth order polynomial; (**c**) pieces of the internal border of the polyurethane layer obtained after filtration (Figure 9 (6)) and thresholding (Figure 9 (7)); (**d**) polyurethane internal border approximated using the spline function; (**e**) thickness map of polyurethane cover lamina in 3D view; (**f**) flat coloured thickness map of the polyurethane cover layer.

**Figure 11:**
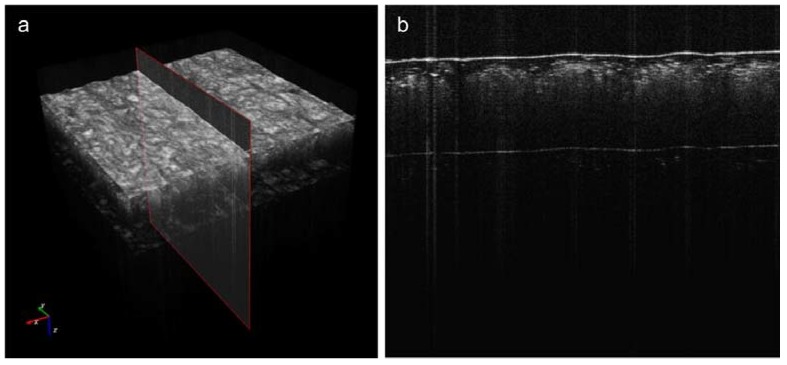
Example OCT *C*-scan of a Cordura 4 (Camouflage, bottom rows of Figure 2 and Figure 5) sample region *R* (**a**) with selected *B*-scan in the YZ-plane (**b**).

**Figure 12:**
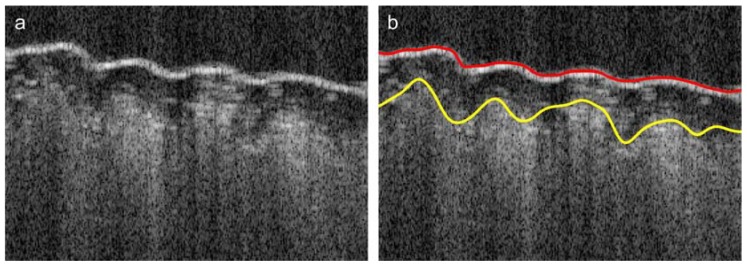
Illustration of selected stages in the composite image segmentation workflow presented in Figure 9. (**a**) the *B*-scan selected from an OCT image; (**b**) the results of composite surface and internal layer boundary detection inside the selected *B*-scan, visible as red (upper) and yellow (lower) lines, respectively.

(3) K(u,v,w)=G(u,v,w)·L(u,v,w)

(4) $$G\left(u,v,w\right)=\frac{1}{S_{G}}\left(-\frac{u^{2}}{2\sigma_{x}^{2}}-\frac{v^{2}}{2\sigma_{y}^{2}}-\frac{w^{2}}{2\sigma_{z}^{2}}\right),\;L\left(u,v,w\right)=\frac{u^{2}}{2\sigma_{x}^{4}}+\frac{u^{2}}{2\sigma_{x}^{4}}+\frac{u^{2}}{2\sigma_{x}^{4}}-\frac{1}{\sigma_{x}^{2}}-\frac{1}{\sigma_{x}^{2}}-\frac{1}{\sigma_{x}^{2}},$$

where I(x,y,z) and J(x,y,z) are as before; K(u,v,w) denotes the LoG filter kernel limited in a cuboid [U×V×W]; G(u,v,w) and L(u,v,w) are Laplacian and Gaussian kernel components; and $\left[\sigma_{x}^{2},\;\sigma_{y}^{2},\;\sigma_{z}^{2}\right]$ is the Gaussian smoothing variance parameter. Additionally, $S_{G}=\sum_{u,v,w}G\left(u,v,w\right)$ and $S_{K}=\sum_{u,v,w}G\left(u,v,w\right)L\left(u,v,w\right)$ stand for normalizing factors. The OCT image filtering sequences $F_{1}\left(\cdot \right)$ and $F_{2}\left(\cdot \right)$ correspond to the pipe $MF\left(P,Q,R\right)|LoG\left(U,V,W,\;\sigma_{x}^{2},\;\sigma_{y}^{2},\;\sigma_{z}^{2}\right)$ (Figure 9 (1), Figure 9 (6)), with different local region sizes [P,Q,R] or [U,V,W] and Gaussian smoothing variances $\left[\sigma_{x}^{2},\;\sigma_{y}^{2},\;\sigma_{z}^{2}\right]$, which are experimentally adjusted to effectively extract the composite layer boundaries. The algorithm thresholding steps (Figure 9 (2), Figure 9 (5)) also use the experimentally selected thresholds $T_{1}$ and $T_{2}$ to obtain composite surface voxels $B_{1}\left(x,y\right)$ or polymer layer internal boundary voxels $B_{2}\left(x,y\right)$, as shown in Equations (5) and (6), respectively.

(5) $$B_{1}\left(x,y\right)=min_{z}\left(I\left(x,y,z\right)>T_{1}\right)$$

(6) $$B_{2}\left(x,y\right)=min_{z}\left(I\left(x,y,z\right)>T_{2}\right),\;z>z_{1}+dz_{1},$$

where $dz_{1}$ is the algorithm parameter. The material surface boundary may fade or even disappear at different lengths (see Figure 11b, Figure 12a), with no detectable bottom edges close to the surface. The surface boundary values $B_{1}\left(x,y\right)$ in Equation (5) may therefore be undefined at some (x,y) positions, where the boundary voxel is not found at an acceptable depth. These considerations apply even more to the internal layer boundary voxels *B*_2_(*x*, *y*) in Equation (6), which are located below the approximated composite surface—i.e., $z\left(x,y\right)>z_{1}\left(x,y\right)+dz_{1}$. The flexible composite material was partially stretched using micrometer table holders to smooth its surface. Therefore, the surface can be initially approximated by a least-squares fit of the 2D data $B_{1}\left(x,y\right)$ with the fifth order polynomial (Figure 9 (3)). This approximation task uses the Matlab Central functions polyfitweighted2 and polyval2, shown in Equation (7), which are available in [41]. The undefined data values $B_{1}\left(x,y\right)$ were assigned the weights w(x,y)=0 and other boundary data had the weights w(x,y)=1.

(7) $$p_{1}=polyfitweighted2\left(xr,yr,B_{1},5,w\right),\;z_{1}\left(x,y\right)=polyval2\left(p_{1},xr,yr\right),$$

where xr=[1,2,…,X], yr=[1,2,…,Y] denote Matlab style vectors of all possible *x* and *y* image coordinates, respectively. As can be seen, for example, in Figure 10b, polynomial surface approximation is not very accurate, but provides *z*-coordinates for all surface pixels and may be used to determine and eliminate outlying data $B_{1}\left(x,y\right)$ before the second stage of approximation. The selection condition of the input data for this stage is given in Equation (8) as

(8) $$\left|B_{1}\left(x,y\right)-z_{1}\left(x,y\right)\right|<\Delta z_{1},$$

where $\Delta z_{1}>0$ denotes the algorithm parameter. For accurate approximation of the polymer surface, a fast-smoothing spline method was selected, as described in [42]. The method is based on forward and inverse discrete cosine transform (*DCT*) and can be expressed as an iterative convergent process, as defined in Equation (9).

(9) $$B_{1}^{\left(k+1\right)}=DCT^{-1}\left(\Gamma^{N}{}^{\circ}DCT\left(W_{1}{}^{\circ}\left(B_{1}\left(x,y\right)-B_{1}^{\left(k\right)}\left(x,y\right)\right)+B_{1}^{\left(k\right)}\left(x,y\right)\right)\right),$$

(10) $$\Gamma^{N}=1÷\left(1^{N}+s_{1}\Lambda^{N}{}^{\circ}\Lambda^{N}\right),$$

where N=2 is data dimensionality; DCT and $DCT^{-1}$ denote discrete cosine transform and its inverse, respectively; $W_{1}$ is the weight matrix of $w_{ij}\in \left[0,1\right]$ for $B_{1}\left(x,y\right)$; $\Gamma^{N}$ is a diagonal matrix of nonzero values; the symbol ° denotes the Schur product; and the symbol ÷ is element by element division. $\Lambda^{N}$ is the following *N*-rank tensor:

(11) $$\Lambda_{i_{1},i_{2},\ldots ,i_{N},}^{N}=\sum_{j=1}^{N}\left(-2+2cos\frac{(i_{j}-1)\pi }{n_{j}}\right),$$

where *n*_j_ is image size in dimension *j*. In Equation (10) the symbol *s*_1_ represents a positive smoothing factor, rises in which increase the smoothness of the surface approximation. This method is applied in the case of $B_{1}\left(x,y\right)$ with missing outlier data by assigning zeros to their weights in the matrix *W*_1_. The solution of Equation (9) is obtained when the specified approximation tolerance or maximal number of iterations has been reached. The smoothing defined in Equation (9) is performed by implementing the Matlab function cited in Equation (12).

(12) $$z_{1}\left(x,y\right)=smoothn\left(B_{1},W_{1},s_{1}\right),$$

where *B*_1_, *W*_1_, *s*_1_ have the same meaning as in Equation (9) ÷ Equation (11). To improve internal boundary approximation, its voxels $B_{2}\left(x,y\right)$ obtained according to Equation (6) are coarsely approximated with the fifth order polynomial, according to Equation (13).

(13) $$p_{2}=polyfitweighted2\left(xr,yr,B_{2},5,w\right),\;z_{p}\left(x,y\right)=polyval2\left(p_{2},xr,yr\right)$$

Further approximation is limited by the difference $\left|z_{p}\left(x,y\right)-B_{2}\left(x,y\right)\right|<\Delta z_{2}$ removing marginal data. In Equation (13), the weights w(x,y)=0 for the ignored subset of boundary data $B_{2}\left(x,y\right)$. The composite internal boundary is hardly visible and almost disappears in some image regions (see Figure 12a,b). Binarization using the global threshold *T*_2_ (Figure 9 (7)) provides a set of non-uniformly distributed data samples with many gaps, such as those observable in Figure 10c. The process of layer boundary approximation should construct a smooth approximation of the scattered data with expected curvatures not less than those present on the composite surface. This boundary can also be properly approximated in the XY plane using the same spline method described in Equations (9) and (12). In this case:

(14) $$z_{2}\left(x,y\right)=smoothn\left(B_{2},W_{2},s_{2}\right),$$

where *W*_2_ represents a weight matrix for the data *B*_2_ with binary values equal to 1 or 0, for left or missing (outlier) data, respectively. The smoothing factor *s*_2_ value is adjusted experimentally, but is typically greater than *s*_1_ from Equation (12). It is correlated with *B*_2_ data variance around its polynomial approximation. Finally, the thickness map d(x,y) of a composite cover layer (Figure 10e–f) is computed as the difference between the layer internal boundary $z_{2}\left(x,y\right)$ and the surface boundary $z_{1}\left(x,y\right)$ in the X×Y domain, as given in Equation (15).

(15) $$d\left(x,y\right)=z_{2}\left(x,y\right)-z_{1}\left(x,y\right),\;x\;\epsilon \;\left[0,X-1\right],\;y\;\epsilon \;\left[0,Y-1\right],$$

The results derived from the presented algorithms have low sensitivity to variations in the filtering parameters and the parameters for boundary data approximation. These parameters were adjusted experimentally only once for the tested series of composites and remained unchanged in all cases.

## 4. Results and Discussion

Experimental validation of the proposed method for measuring the polymeric layers of textile composites was performed using four Cordura samples (categories) with coverings of different thicknesses. The tested categories are detailed in Table 1. Layer thickness maps of each of the images were generated using the algorithm described in Figure 9, which was executed in the computing environment described in Section 2.4.

The algorithm parameters used for the series of measurements are as follows:

- [P,Q,R] = [15,15,7] or [3,15,7] voxels—MF filter dimensions in Equation (1) respectively for $F_{1}\left(\cdot \right)$ or $F_{2}\left(\cdot \right)$ boundary filtering,
- U,V,W = [15,15,13] or [3,15,13] voxels—LoG filter dimensions in Equation (3) respectively for $F_{1}\left(\cdot \right)$ or $F_{2}\left(\cdot \right)$ boundary filtering,
- $\left[\sigma_{x},\sigma_{y},\sigma_{z}\right]$ = [5,5,5] voxel—standard deviations of Gaussian smoothing in LoG filter used in Equation (4),
- $T_{1}=0.5÷0.6$, $T_{2}=0.4÷0.5$—thresholds for the normalized boundary image I(x,y,z) in Equations (5) and (6), respectively,
- $zmax_{1}=50$ voxels, $zmax_{2}=250$ voxels—initial z-limit for searching the surface and internal boundaries,
- $dz_{1}=15$ voxels—distance from the approximated surface at which to start searching for the internal boundary,
- $s_{1}=500$, $s_{2}=200÷500$—the smoothing factors in Equations (12) and (14), respectively,
- $\Delta z_{1}$ equal to the doubled standard deviation of $B_{1}\left(x,y\right)-z_{1}\left(x,y\right)$ in Equation (8),
- $\Delta z_{2}=20$ voxels—the limit for corrected searching for the internal layer boundary.

The set of parameter values was selected only once for each of the tested Cordura categories.

**Figure 13:**
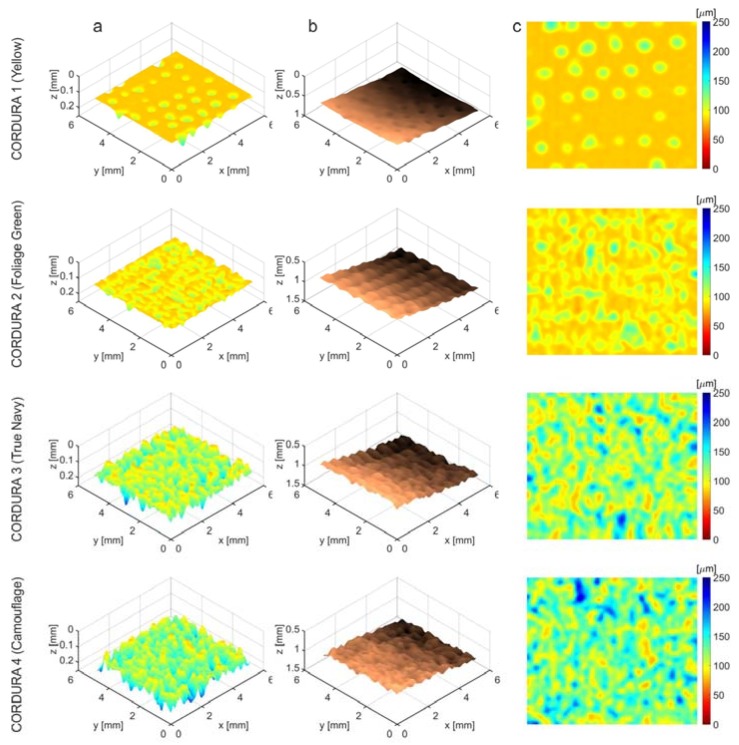
Exemplary thickness maps of the polyurethane layers in different Cordura samples (categories): (**a**) perspective view; (**b**) internal boundary depth; (**c**) XY-plane).

Exemplary thickness maps for single regions of each Cordura type are displayed in Figure 13 as a mosaic. The rows show single scans of each of the tested material types; the columns a, b and c provide different views of the scanned regions. Column c contains two-dimensional XY thickness maps of the scanned regions, expressed in the same depth scale from zero to 250 μm, matched to the different ranges for cover layer depth in the three Cordura categories. The same maps with perspective views are shown in column a. Irregularities in the internal borders of the cover layers are visualized in the images in column b using a copper colour map. 

**Figure 14:**
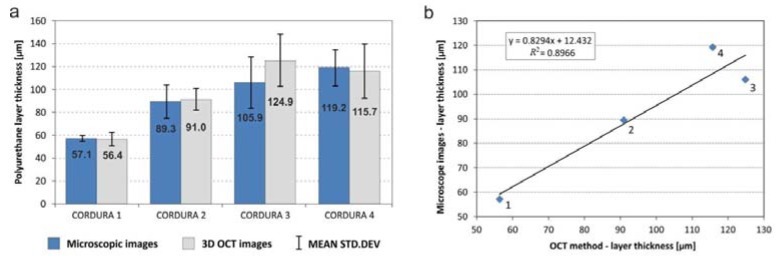
(**a**) Mean values of polyurethane lamina thickness for four types of Cordura obtained using a manual method from microscope images and automated measurements of OCT images; (**b**) The results had a strong relationship, with a coefficient of determination of $R^{2}=0.8966$, b and a Pearson correlation coefficient of r=0.9844.

**Figure 15:**
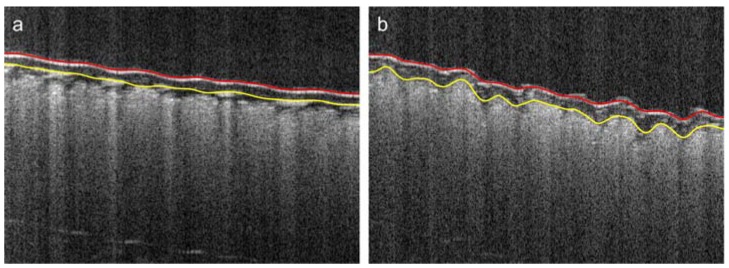
Example Cordura *B*-scans for two types of polymer layer: (**a**) Cordura 1 (yellow) with detectable internal boundary of the laminated polymer layer; (**b**) Cordura 3 (true navy) with polymeric internal boundary adhering to the fabric layer.

The mean thicknesses and standard deviations of the cover layers of the tested Cordura samples (categories) were averaged over the 10 scanning regions, as described in Section 2.4. The values are presented in Figure 14a as a plot of bars grouped in pairs. The left-hand components of each pair refer to microscopic measurements, as described in Section 2.2. The right-hand components refer to results for the same materials calculated from OCT images. In the case of laminated Cordura 1, the internal boundary of the polymer was partially visible in *B*-scans and could be successfully detected, as illustrated in Figure 15a. 

In other cases, the applied polymer diffused into the fabric layer and the boundary of its layer adhered to the fabric, as shown in in Figure 15b. According to the OCT image measurements, laminated Cordura 1 had both the thinnest polymer layer, with a mean thickness of 56.4 μm, and the lowest averaged standard deviation, at 5.8 μm. The reason for this is the relative evenness of the layer of laminated polymer, which does not penetrate between the strands of the nylon fabric. The sample of Cordura 3 (true navy) had the highest average polymer thickness, at 124.9 μm, with an average standard deviation of 22.7 μm. The plot in Figure 14b shows a significant correlation between the average depths of the polymer layer measured using microscope images and those obtained from 3D OCT images for different Cordura categories. The correlation is expressed by both $R^{2}=0.8966$ and the Pearson coefficient r=0.9844.

Both partial times for executing various stages of the algorithm and the total execution time are provided in in Table 2. All the times are average values from calculations for 10 scanning regions selected from each composite sample. With GPU parallel processing (described above), border filtering of around 200 million voxels for a scanned region required on average only $\bar{\tau_{1}}\approx 22\;s$ in the first step (Figure 9 (1)) and $\bar{\tau_{3}}\approx 7\;s$ in the sixth step of the algorithm (Figure 9 (6)), when smaller size filtering was applied. This can be verified in the algorithm parameter list above. Three-dimensional thresholding, and polynomial and spline approximation of the air–material boundary required only around 2.3% of the averaged total run time $\bar{\tau_{0}}$. Similar operations for the internal cover layer boundaries took significantly longer ($\bar{\tau_{4}}\approx 50\%\;\bar{\tau_{0}}$), because this edge is weakly visible and therefore harder to detect and smooth.

## 5. Conclusions

Microscopic measurement of the polyurethane layer in textile composites requires complex and time-consuming preparation of samples, which must be sliced with precision from the tested fabric and held appropriately in the visual field of the microscope before the cross-section can be analyzed. In contrast, OCT imaging and measurement is a non-invasive technology that does not require any sample preparation. The results presented in this paper show a high correspondence between measurements using optical microscopy and those obtained by applying the OCT imaging method in the case of Cordura composite. Visual inspection using OCT enabled the Cordura materials to be distinguished in terms of the presence and thickness of the polyurethane surface layer. The limitations of the presented method are that the maximum depth of the investigated layer was about 3.5 mm, the translucency of the tested polymer surface layer, and the limited area (5mm × 5mm) of the depth maps obtained during one scanning.

In future studies, the authors plan further material tests using OCT imaging, to determine the functional relationship between the thickness of the polyurethane layer and the strength of the electro-conductive metal layers applied to composite substrates by vacuum deposition.

## Figures and Tables

**Table 1:** Characteristics of Cordura samples.

Cordura No.	Trade Name	Manufacturer	Surface Weight [g/m^2^]
Cordura 1	Yellow (unknown serial number)	Miranda Ltd. Turek, Poland	195
Cordura 2	CTD1000MS—Foliage Green	Rockwoods Ltd. Loveland, CO, USA	380
Cordura 3	CTD1000—True Navy	Rockwoods Ltd. Loveland, CO, USA	365
Cordura 4	Camouflage (unknown serial number)	Miranda Ltd. Turek, Poland	460

**Table 2:** Execution times for various algorithm stages in the Matlab environment averaged over 10 sample regions for each Cordura sample. $\tau_{1},\tau_{3}$—the times of surface and internal boundary filtering, respectively (Figure 9 (1), (6)); $\tau_{2}$—the time of surface boundary thresholding and approximation (Figure 9 (2) ÷ (5)); $\tau_{4}$—the time of internal boundary thresholding, approximation and thickness computing (Figure 9 (7) ÷ (11)).

Cordura Type	$\tau_{1}$ [s]	$\tau_{2}$ [s]	$\tau_{3}$ [s]	$\tau_{4}$ [s]	$\tau_{4}$ [s]
1	22.36	1.14	7.14	15.86	46.50
2	22.40	1.65	6.72	46.66	77.41
3	22.37	1.53	6.73	35.26	65.88
4	21.67	1.20	7.01	23.71	53.89
